# “Don’t Even Smoke But I’ll Buy That” Twitch as a New Venue for E-Cigarette Promotion

**DOI:** 10.3389/ijph.2024.1607881

**Published:** 2025-01-20

**Authors:** Hüseyin Küçükali

**Affiliations:** ^1^ Centre for Public Health, Queen’s University Belfast, Belfast, United Kingdom; ^2^ Research Center for Healthcare Systems and Policies, Istanbul Medipol University, Istanbul, Türkiye

**Keywords:** tobacco, vaping, electronic cigarettes, social media, influencer, commercial determinants of health

## Abstract

**Objectives:**

This study examines the covert promotion of e-cigarettes, specifically Juul, on the video live-streaming platform Twitch, focusing on the content and platform-specific dynamics that may influence its effectiveness.

**Methods:**

This qualitative case study of a non-gaming Twitch stream included data from influencer conversations, viewer comments and visual elements. Thematic analysis, coding, and categorisation were conducted using ATLAS.ti.

**Results:**

The livestream, which attracted over ten thousand viewers, featured three friends vaping and discussing their personal experiences with Juul. Themes included curiosity, device sharing, and smoking cessation benefits. The stream fostered a casual, engaging atmosphere, with viewers interacting extensively with the influencers and also other viewers. Despite suspicions of industry sponsorship, viewers reacted positively and even joked about the sponsorship. Several viewers, including those who claimed not to smoke, expressed interest in Juul.

**Conclusion:**

The study highlights concerns about the promotion of harmful commodities on Twitch, where strong community engagement, monetary incentives, and a lack of specific content policies create a conducive environment for such practices. This underscores the need for greater scrutiny and regulation of e-cigarette promotions on the platform.

## Introduction

In their pursuit to entice new generations into nicotine addiction, tobacco companies have extensively used influencer marketing tactics [1]. Their activities on popular social media platforms such as Twitter, Instagram and YouTube are well documented in literature [2, 3]. Previous studies identified various strategies used for e-cigarette promotion on social media from featuring product characteristics to building pro-vape communities [3]. It is also shown that exposure to marketing in media is associated with an increased likelihood of e-cigarette use among middle and high school students [4]. Although some government authorities aim to hold companies accountable for influencer marketing, sponsorships are rarely disclosed [3].

The social media landscape continuously evolves. New platforms focused on video live streaming become more and more popular. Leading in the video live-streaming sector, Twitch was founded in 2011 and later acquired by Amazon. It was originally designed for gaming content but later introduced a category known as In Real Life (IRL) which refers to a wide range of non-gaming content including “just chatting,” cooking, or walking around. With 16% of average concurrent viewers, “Just Chatting” became the category watched more than any other game individually [5]. As of December 2023, Twitch has 2.5 million average concurrent viewers [5], 41% of whom are 16–24 years old [6].

A short paper reported an e-cigarette promotion during gameplay on Twitch [7]. However, tobacco promotion in this burgeoning venue remains mostly unknown. This study analyses an example of a non-gaming Twitch stream covertly promoting e-cigarettes, specifically Juul, to document the content of tobacco promotion and to understand platform-specific dynamics that may influence its effectiveness.

## Methods

In this qualitative case study, we employed the thematic analysis method [8] to document and interpret a single Twitch stream featuring three influencers modelling and promoting e-cigarette usage.

We identified the Twitch influencer from a picture he shared on another social media platform that shows him vaping. He was one of the most popular Turkish streamers, largely streaming IRL videos. Following his channel on Twitch we were able to observe a few instances where he displayed usage of an e-cigarette, Juul. However, one particular stream in July 2019 stood out regarding e-cigarette promotion.

The live stream and its recording were publicly available. We took observation notes during the stream using a semi-structured form. The observation form included: what is shown on the screen, what is said (about e-cigarettes), how it is said, and how it is received by the audience. We transcribed the conversations verbatim to create textual data. We took screenshots from the video to analyse visual elements. We also extracted relevant comments from the chat box beside the stream containing reflections of live viewers. Data from these four sources was imported to ATLAS.ti software for analysis. The software is used only for the organisation (i.e., manual labelling and categorisation) of the information by the researcher.

Although the stream was already in the public domain, we used several strategies to conceal identifiable information when reporting the results. We replaced influencers’ names with pseudonyms, rounded their follower counts (Table 1), generated anonymised reproduction of the screenshots using ChatGPT 4 and removed the usernames of the viewers.

**TABLE 1:** Approximate number of followers of influencers on various social media platforms (Türkiye, 2019).

	Twitch (n)	YouTube (n)	Instagram (n)	Twitter (n)
Eren	1,000,000	200,000	600,000	140,000
Hakan	None	3,000,000	1,000,000	20,000
Onur	None	900,000	700,000	80,000

Data was coded inductively focusing on the promotion of e-cigarettes and community dynamics. Codes were grouped to form categories and themes. Findings from viewer comments were used to triangulate findings from the influencer conversations. Observation notes were used to integrate viewer comments and visual elements into themes from influencer conversations. Direct quotations are provided for each theme to support the findings. Because the stream was in Turkish language, quotations were translated by the researcher.

## Results

Here we present study findings beginning with the description of the setting as it appears on the screen and summarizing the backstory told by influencers as their journey into using e-cigarettes. Then we explain the main themes that have been identified and support them with illustrative quotations from both influencers and viewers.

The stream commenced at 9 PM and was 2 h and 40 min long. Over ten thousand viewers joined live, and more were able to watch it later [9]. Together with the original streamer, two other social media influencers were present in the stream as guests. Table 1 shows the approximate follower counts of each of the three influencers on various social media platforms to give an idea about their activity and outreach.

### The Setting: Just Chatting in a Living Room

**FIGURE 1:**
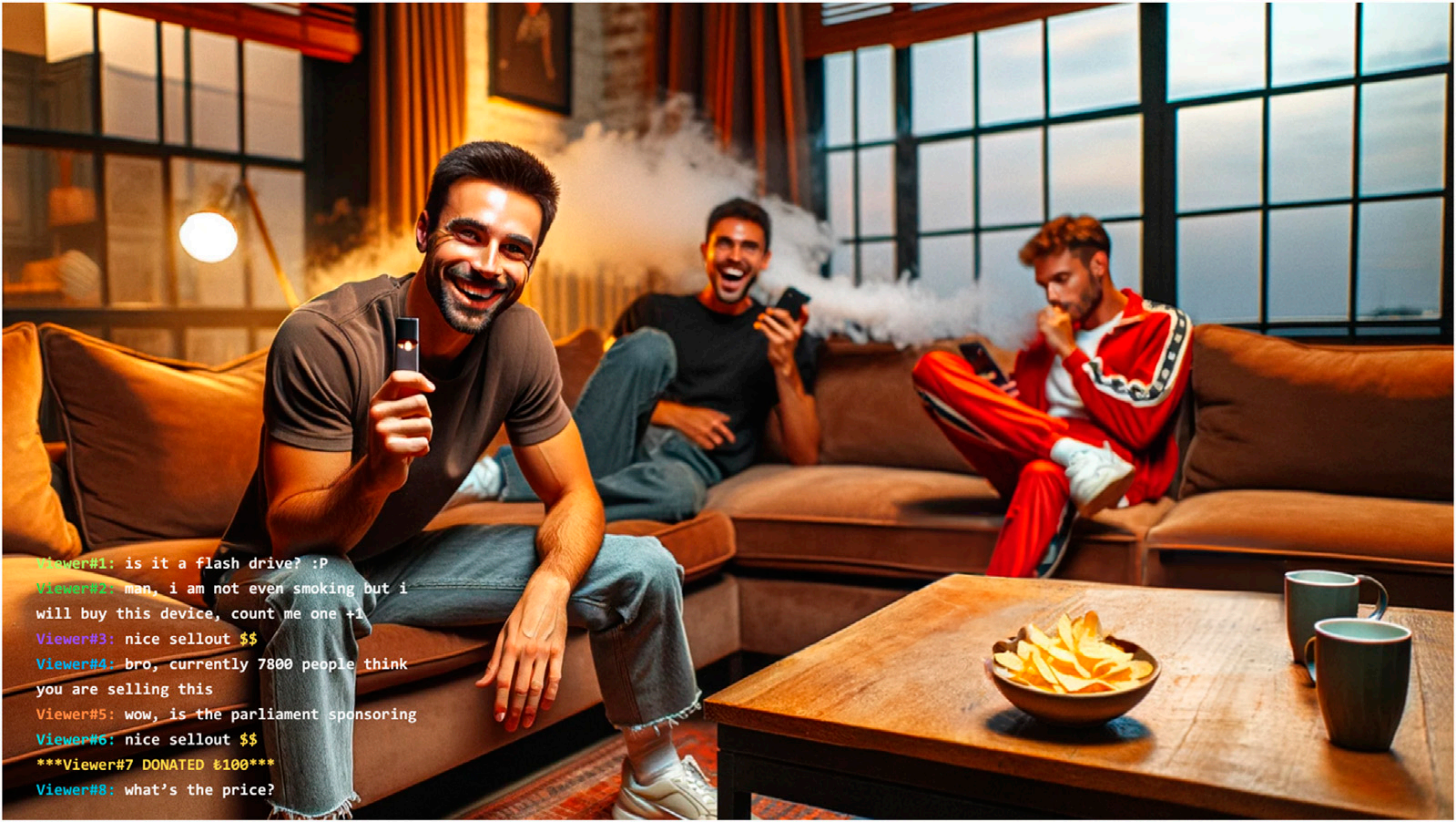
An anonymised reproduction of the appearance of the Twitch stream that promotes electronic cigarettes with a “chat” section in the bottom left corner including viewer comments (Türkiye, 2019).

The setting was a cosy living room where three friends were relaxing on a sofa, chatting, drinking, and joking with each other. The scene creates an impression that the viewer is sitting right next to them as their fourth companion. Occasionally, each talked directly to viewers and impersonated them by referring to them collectively as “Chat”. They responded to some of the comments which never stopped from the beginning to the end. They were using slang and profane language. During the stream, they kept vaping and sharing each other’s vaping devices. Figure 1 illustrates the setting of the stream.

The stream was publicly accessible and there was no restriction to prevent minors from watching it. Although it was not possible for us to confirm their ages, the language of their comments (e.g., mentioning the influencer as “[big] brother”) suggested the presence of underage viewers.

### A Vape Story

Particularly 15 min were dedicated to a story promoting e-cigarettes, namely, Juul. The outline of the story was as follows based on observation notes:

Onur used to smoke, especially menthol cigarettes. But he does not like the look of a smoker. He quit smoking a year ago. Later, a friend brought him a Juul from abroad and he started vaping. While working, Onur was vaping between times. Eren was smoking heavily back then. He was distressed because he could not smoke in the workplace. Eren got curious about Juul and tried vaping from Onur’s device. Hakan never smoked but was also wondering about Onur’s fancy device. “A friend” of Onur delivered Juul devices and supplements to Eren and Hakan for a fee – illegally. Eren quit smoking. Hakan gifted his device to their boss, yet he kept vaping from his friends’ devices because he liked vaping. It turns out that the one that he likes the most is the one containing nicotine.

### Information on E-Cigarettes

“Don’t tell it half-heartedly! There’s a device called Juul. For those who don’t know, it is an electronic cigarette…” – Hakan

While telling this story they provided detailed information on e-cigarettes. This information involved what it is, where to find it, how much it costs, how long it lasts, its components, and consumables like liquids and pods. They also demonstrated how to use, refill, and charge the device.

Eren shares his amazement “Look how interesting it is. They send you a flavour catalogue over WhatsApp. It says Juul starter pack! Numerous options in the catalogue, there is chocolate, there is tobacco something … You can choose even the percentages.” Onur adds “There are 1.7 milligrams or 5.5 milligrams nicotine [options] etc.” Later he refills a pod and introduces it to friends “This one has aromatherapy notes. It stops capillaries in your brain!… Bro, it is Virginia Tobacco!”

Viewers asked mostly about the price of the Juul (n = 13) and a few viewers (n = 3) mentioned flavours and liquids.

### Benefits of E-Cigarettes

“People may think I’m trading this. Dude, I’m helping one of you quit smoking, trying to prevent the other from starting.” – Onur

The main benefit of the Juul emphasized over and over during the stream was smoking cessation. Eren used to smoke 3 packs/day and “never smoked since the day he got [the Juul].” Onur adds “Eren was using it for 2 weeks. Eren’s life was a ten [out of ten]!”. Onur warns viewers “Don’t smoke. Don’t get bad habits.” But if you somehow got into it, you could use e-cigarettes instead. Scorning other methods, he says “Dude, if you can, go quit via meditation or acupuncture. This is my way, man, I don’t want to walk around with needles in my ears.” Even if you already quit, you need something to substitute the habit according to him, “Your hands will fidget. You either spin a rosary, or that cigarette must come and go.”

To contrast with e-cigarettes, Onur expressed disgust against traditional cigarettes as follows: “I don’t want to stink,” “your mouth your moustache become like shit.”, “You will be disgraced. your hand your lips, your teeth, your moustache turn yellow.”, “you don’t enjoy the food you eat.”

Another advantage implied was that one can vape in public spaces. Eren was “getting mad” because he couldn’t smoke in the workplace while Onur “was puffing in every break.” “It makes you wonder” Eren adds.

Just a few viewers send opposing messages. “He seems like not knowing this (the e-cigarette) is even worse. Should we enlighten him?” one asked. Another said, “Don’t use this shit.” These messages are lost among hundreds of conforming messages.

### Curiosity and Device Sharing

“Normally, I do not smoke. I didn’t want to get into such damn things. But while I am with you, while you are sucking (vaping) I fancy to suck, dude.” – Hakan

Onur described Juul as his “dream device.” During the story, Juul is an object of curiosity, both for people who do (Eren) and do not (Hakan) smoke. They saw it and wanted to vape. Onur asked Hakan to elaborate on why he fancies, he said “It looks cool and it smells nice!”. It is portrayed as a technological product “like a USB” (flash drive) and might be a good gift for friends.

“Man, I am not even smoking but I will buy that device, count me one.” – A viewer

Many viewers got curious about this technological gadget and asked (some genuinely some jokingly) if it was a flash drive (n = 52) in the chat. “[Eren,] do you addict these kids to a USB?”. Some expressed their interest in buying Juul (n = 5) and one asked the host to start a Juul giveaway.

Linked with curiosity, sharing the Juul was a significant theme in the stream. They kept using each other’s devices during the stream, but they also talked about it several times. They shared their devices whenever someone else fancied to try. Eren said his device goes around among the people in the workplace. “You know who I took it back from. Man, it has travelled from hand to hand, to a director. It started from Hakan!” he adds “Bless lucky stars if we didn’t get a disease.” Contradictory though they also mention this act with some abomination “foul guy,” “I don’t know how many bacteria are in your mouth.” However, it did not stop them from sharing the Juul.

### Joking on Sponsorship

“I swear, if he’s not selling this, then I’m nothing, man!” – Eren

Each of the three influencers has a track record of sponsorships with multinational companies from industries ranging from sugar-sweetened beverages to cosmetics, and fast fashion. Most of them were targeted at young consumers. However, it was not clear if this particular stream was somehow sponsored by tobacco companies or e-cigarette vendors. Nevertheless, it was the most common theme that joked about an obscure sponsorship. While Onur explained the benefits and the experience of the Juul, others frequently interrupted and ascribed having an interest to him.

“How much did this guy get paid for this?” – Eren

“Explain to us now how much commission you’ve received.” – Hakan

“… then you got into Juul business, ha?” – Hakan

“He is almost to say, buy two get one free” – Eren

Arguably, the act of joking might have a normalizing effect on tobacco sponsorship. It may position tobacco sponsorship similar to any sponsorship which is customary in streaming.

“Bro, currently 7,800 people think you are selling this!” – A viewer

We counted 55 viewer messages implying a sponsorship like “Nice sellout!”, “He must’ve coined the money” and numerous money emojis. Some followers seem to be even excited about the possibility of a big sponsor: “Wow, is the Parliament sponsoring?”. Two viewers got fed up with e-cigarette promotion and asked, “When will ads end?”.

### Engaged and Interacting Community

The live chat feature engaged the viewers into the conversation in streaming which is unparalleled among other social media. We observed that the viewers were watching the stream with high attention. It was not possible to keep the count of comments manually. Here we only quoted the most relevant comments. However, to give a rough idea, they were sending about 3–30 comments per second, half of which were not legible. They were racing to catch funny phrases from the conversation, identify brands of objects (e.g., clothes, bags) that got into the scene, and write them back in the chat box. The host (Eren) asked for small tasks from his followers such as taking pictures or providing some information as well as moderating the chat flow. Donations from the viewers kept coming during the stream showing their support. Each donation is announced, automatically, through big animations on the screen and celebrated in the chat.

Viewers were not only interacting with the streamer but also with other viewers through the chat. This enabled self-organising dynamics. For example, independent from the streamer they organized two different ways of obtaining e-cigarettes. One said that Juul is sold for 50 euros in France and others asked him to buy for them. Another viewer pointed to a name who was known to the community, to obtain (imitation) e-cigarettes from for a lower price.

## Discussion

This study documents an example of covert e-cigarette promotion on a streaming platform, Twitch. It is unknown whether the steam is sponsored by the industry, but e-cigarette promotion is evident.

The studied case provides insights into the themes of the e-cigarette conversation and the dynamics of the online community exposed to it. Although they made a case for the e-cigarette helping one of the influencers quit smoking, what actually happened was two others started vaping nicotine; one already quit smoking a year ago, and one never smoked before. However, this remained unnoticed amongst laughter. Thousands of viewers are introduced to Juul. Although they suspected the stream to be sponsored, they joked about it along with the influencers themselves. It was not difficult to raise curiosity for Juul in such an engaged community. Some viewers, including people who claimed to not smoke, expressed their interest in buying Juul. They even self-organised to plan ways to obtain Juul illegally. Such responses from the community enabled us to observe the influence of the stream on at least the intention to buy e-cigarettes.

A recent review has listed common strategies used for e-cigarette promotion in social media [3]. The current study was able to identify similar strategies in Twitch including information on product characteristics and flavours, using celebrities/influencers, as well as youth-appealing themes and health benefits. Further, the study documented modelling e-cigarette use and device sharing between friends.

**FIGURE 2:**
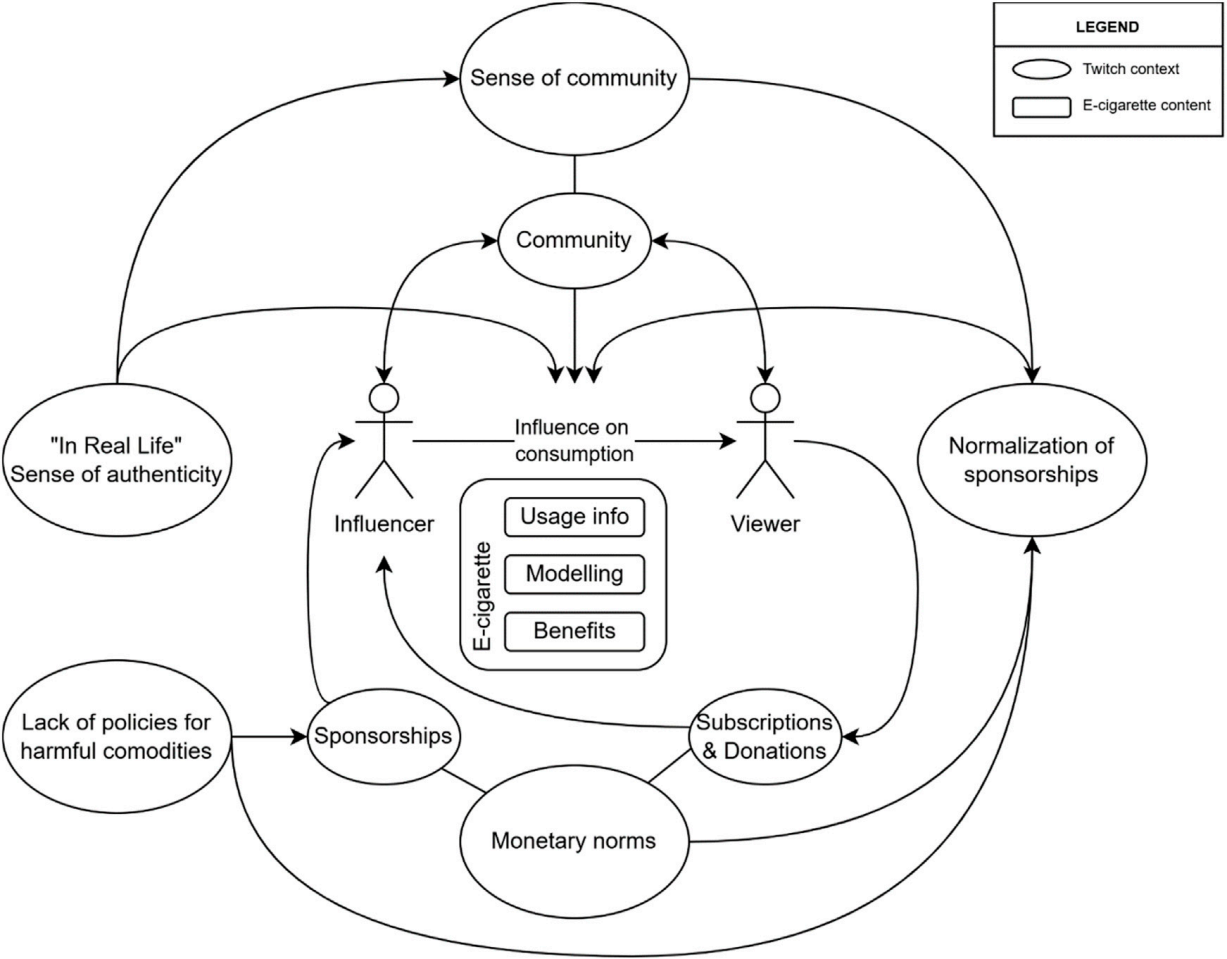
The diagram summarising study findings on e-cigarette content within platform-specific contextual features of Twitch (Türkiye, 2019).

Although strategies used to promote e-cigarettes were not very different from what has been found on other social media platforms [2, 3], the medium, Twitch, has unique qualities that seem to influence how the message is perceived. First, it arguably creates a stronger sense of community and engagement than any other platform [10, 11]. Given the age distribution of its users, it is possible that the platform draws advantage from adolescents’ need to belong. This is especially worrisome considering that Twitch’s content policy lacks specific restrictions for the promotion of harmful commodities such as tobacco [12]. Second, IRL videos are meant to show the genuine lifestyle of the influencer. So, they may be more influential than an ordinary advertisement in which the influencer would take place. Third, money is central in Twitch streams. Paid subscriptions and donations take up a large space both on the screen and in the discourse. Both influencers and followers got accustomed to sponsored content thus potential sponsorship of a harmful industry didn’t ring alarms but instead laughter. Figure 2 summarizes study findings on e-cigarette content within contextual features of Twitch. We urge future researchers to pay closer attention to these platform-specific features. Not only to prevent the industry’s activities but also to utilise such properties of streaming platforms for health promotion and activism [11].

Live streaming platforms pose a challenge to tobacco control as it is very difficult to conduct surveillance of over 2 million hours of videos per day [5] or enforce bans on videos which will disappear in 7–14 days [9] having already reached most of its audience. Taking that into account we recommend 1) imposing restrictions on content policies of streaming platforms 2) application of machine learning techniques for detection and labelling, 3) preventing children’s access, 4) demonetising tobacco-promoting content, and 5) mandating disclosure of sponsorships both by tobacco companies and the influencers.

This case study is meant to be just a call for more comprehensive studies and early preventive actions for tobacco promotion in streaming platforms. Several limitations of this study should be noted, including the reliance on a single Twitch stream for data collection, which might not represent the entirety of e-cigarette promotion on the platform. Moreover, as with any thematic analysis, subjective interpretation and the researcher’s own biases might have influenced the coding process. Data analysis has been carried out by a single researcher due to limited resources. Still, the findings were extensively supported with direct quotations and were mostly aligned with established knowledge from other social media platforms with a few additional suggestions for platform-specific mechanisms. Lastly, although watching the stream was open to anyone, writing in the chat was restricted to paid subscribers (n = 1,151) who might be more engaged than the other ten thousand concurrent viewers.

### Conclusion

Twitch, a popular live-streaming platform, has emerged as a new frontier for behavioural health. This study exposes a concerning trend, the subtle promotion of e-cigarettes on Twitch which is particularly problematic because of its reach among adolescents. Unlike traditional tobacco advertising, these promotions cleverly leverage the unique platform features designed to increase its influence. The strong sense of community cultivates a sense of belonging, making viewers more receptive to influencer marketing. The “In Real Life” format portrays e-cigarette use as a seamless part of a trendy lifestyle, potentially more persuasive than staged advertisements. Furthermore, the constant presence of monetisation through subscriptions, donations and sponsored content, potentially desensitises viewers against industry sponsorships of harmful products like e-cigarettes. These factors combine to increase the vulnerability of viewers to e-cigarette use and normalise this risky behaviour. In order to tackle this problem, a comprehensive approach is required. Streaming platforms like Twitch should implement stricter content policies that explicitly prohibit the promotion of tobacco products including e-cigarettes as well as other harmful commodities. Machine learning algorithms can be used to detect and flag such content. Age verification measures and access restrictions for underage viewers can support risk mitigation. Finally, demonetising content that promotes tobacco products can discourage influencers from such practices. In this context, the study recognizes social media companies, not only tobacco companies, as commercial determinants of health. Hence, implementing such measures constitutes upstream interventions for risky health behaviours, addressing the root causes that contribute to the normalization and use of e-cigarettes particularly among adolescents.

## Data Availability

The anonymised data is available from the corresponding author upon request.
